# Juvenile Atlantic sturgeon survival and movement in proximity to an active cutterhead suction dredge

**DOI:** 10.1371/journal.pone.0300489

**Published:** 2024-11-27

**Authors:** Matthew Balazik, Douglas Clarke

**Affiliations:** 1 Engineer Research and Development Center, United States Army Corps of Engineers, Vicksburg, MS, United States of America; 2 Rice Rivers Center, Virginia Commonwealth University, Richmond, VA, United States of America; 3 Clarke Environmental Consulting, Nellysford, VA, United States of America; Makerere University College of Natural Sciences, UGANDA

## Abstract

The Atlantic Sturgeon *Acipenser oxyrinchus oxyrinchus* has suffered population declines throughout its range. Many knowledge gaps exist regarding how to mitigate threats and better inform recovery efforts. This study examined survival of juveniles during their movements through river reaches undergoing channel maintenance dredging operations. During 2019 and 2020, 268 (30-71cm fork length) juvenile Atlantic Sturgeon were captured and released in proximity to an active cutterhead suction dredge at three sites within the James River, Virginia. Juveniles were captured, some presumed feeding, around 95-145m from the dredge in areas that could easily be avoided if the dredge created a stressful environment. No significant trends in catch-per-unit-effort were found when trawl catch was compared to a reference location or when monitoring gill net catch 100m down current of a dredge over a month-long period at one of the sites. Twenty-nine of the 229 gill net captures were implanted with acoustic tags and telemetry was used to track their movements throughout the river. Four telemetered juveniles tagged prior to this project were also detected moving within dredge operations. Cumulatively, tagged juveniles made at least 125 passes of the dredging operations with no evidence of mortality. All tagged juveniles still within the river were detected following the cessation of dredging. The results of this study support that age 1-2yr Atlantic Sturgeon show no avoidance behavior of areas 100m of an active cutterhead suction dredge and move past dredge operations with low-risk of mortality.

## Introduction

Atlantic sturgeon *Acipenser oxyrinchus oxyrinchus* (ATS) is a federally protected species inhabiting the east coast of the Unites States with all six distinct population segments being listed as either threatened or endangered [1,2]. ATS are a long-lived species with a relatively high generation time making recovery from low numbers a slow process [3–7]. Several obstacles to ATS recovery have been identified, with navigation dredging being listed as a potential threat by altering habitat and macroinvertebrate communities [5,6], hydraulic entrainment [8], and impeding migration [9,10]. Research into how sturgeon species might be affected by dredging operations is very limited. Parsley et al. in the Columbia River, Washington, suggested White Sturgeon *A*. *transmontanus* may be attracted to open water sediment placement areas in search of food [11]. Gill net, acoustic and trawl surveys in the St. Lawrence River indicate that juvenile ATS and Lake Sturgeon *A*. *fulvescens* inhabit sediment placement areas less when compared to control sites [12,13]. Reine et al. [9] used active and passive telemetry to examine survival of five juvenile, 65–86 cm fork length (FL) ATS that were transported from over 10km downstream and released beside an active cutterhead suction dredge (CSD) in the James River, Virginia. No evidence of injury, mortality or migratory impairment was observed. In a later study based on telemetry with a position accuracy of 1m, Balazik et al. [10] similarly showed that CSD had no noticeable effect on ATS migrating to spawning habitat in the James River, Virginia.

Hydraulic entrainment of juvenile ATS by hydraulic trailing suction hopper dredges (TSHD) and CSD is a notable concern. Fish swimming capabilities tend to increase with fish length, so smaller fish have less capability to escape hydraulic intakes [14,15]. TSHDs are typically used to dredge harbor entrance channels, whereas CSDs are responsible for a majority of riverine projects. Seasonal and storm-induced shoaling of navigation channels in rivers necessitates periodic CSD operations to maintain safe navigation depths. Juvenile ATS typically spend their first years within natal rivers; therefore, juvenile ATS would likely encounter dredge operations if dredging occurs in their natal river [6,7]. Although the studies cited above provide useful insights, extensive knowledge gaps persist with respect to juvenile ATS behavioral responses and consequent survival rates in the presence of active dredging operations.

Routine maintenance dredging of the James River, Virginia, federal navigation channel occurs annually in areas that juvenile ATS (here treated as 1-2yr), are known to inhabit. To better inform effective dredging project best management practices with respect for protecting sturgeon, the U.S. Army Corps of Engineers (USACE) Norfolk District, the Engineer Research and Development Center, Virginia Commonwealth University (VCU), and Cottrell Contracting Corporation partnered to conduct an investigation of juvenile ATS behavior and survival around an active CSD working in the James River, Virginia. The primary objective was to use telemetry, catch-per-unit-effort (CPUE) and mark-recapture data to estimate survival of juvenile ATS occupying river reaches being actively dredged. A secondary objective was to determine if juvenile ATS show avoidance behaviors in the presence of CSD due to associated sound and sediment plumes.

## Methods

This study fully complied with guidelines set by VCU’s Institutional Animal Care and Use Committee (#AD20127) and the National Marine Fisheries Service endangered species permit (#20314–01). VCU’s Institutional Animal Care and Use Committee permit was provided by VCU Animal Care and Use Program which is accredited by the International Association for Assessment and Accreditation of Laboratory Animal Care with continuous accreditation since August 3, 1966.

### Study area

**Fig 1:**
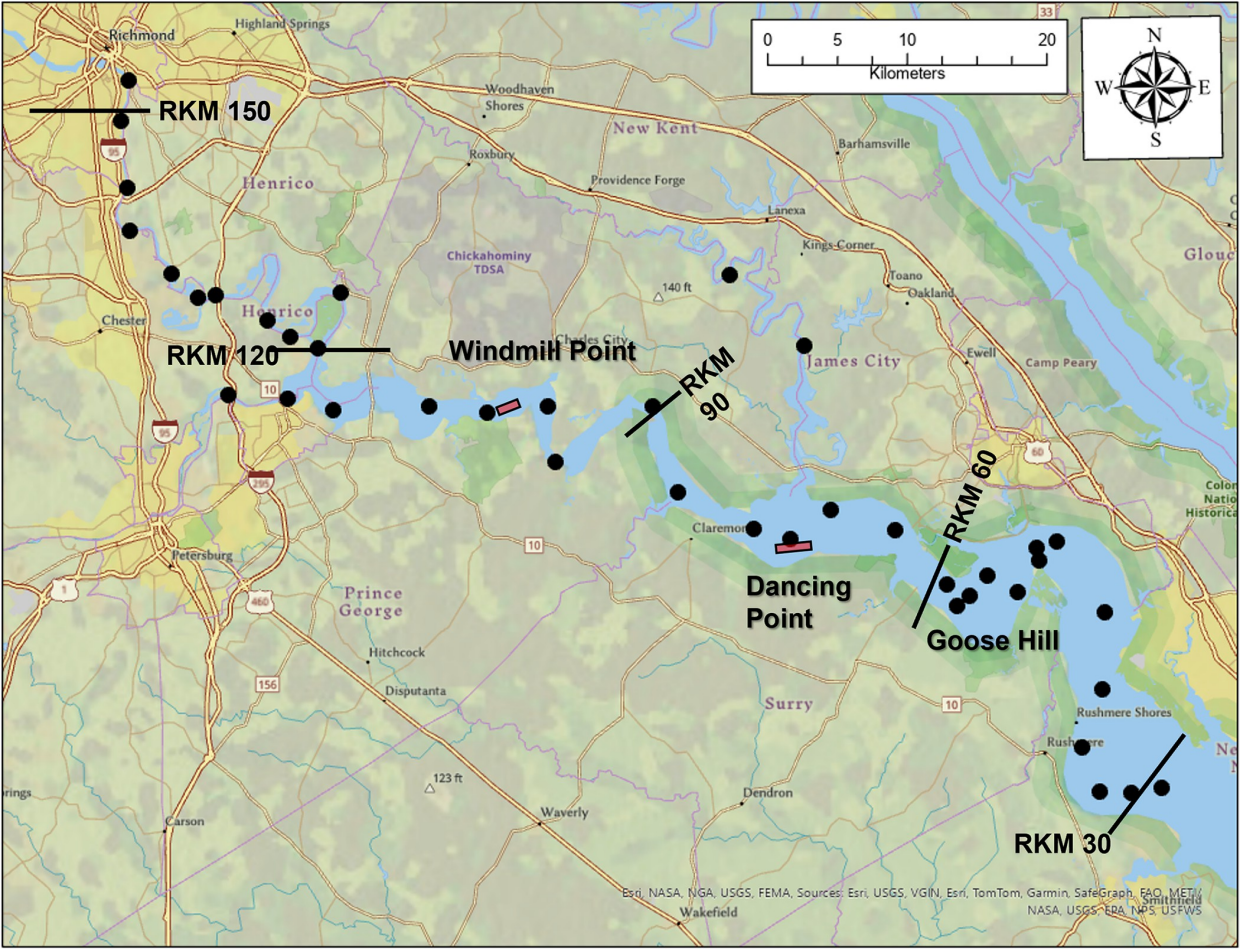
Map showing the three areas (red highlighted rectangles) where juvenile ATS sampling occurred around active cutterhead dredge operations. The black points are passive receiver locations. Some of the passive receivers were damaged or lost during the multiyear tracking period.

This study was conducted in Virginia state waters open to public access and in which do not require permits/permissions to access. Federal regulations state that a 91.4m wide, 7.6m deep channel must be maintained for navigation in the James River, Virginia. The study occurred at three shoaling areas of the tidal portion of the river between river kilometers 45 and 103 (Fig 1). The three shoaling areas are referred to as Dancing Point, Windmill Point and Goose Hill. Due to chronic shoaling, maintenance dredging by a CSD is required at a minimum of one, if not all three locations, annually. For this study the dredging period for Windmill Point was November-December of 2019 while Dancing Point and Goose Hill were dredged September-November 2020.

The CSD *Marion* conducted maintenance dredging at all three study locations. The *Marion* is a 40m long, 8.0m wide, 2.0m draft barge with a 1492kW hydraulic centrifugal pump connected to a 39cm inner-diameter intake pipe located just shipward of the cutterhead. Pipeline discharge velocities were approximately 3.4m/s. All dredged material was placed back into the water via a partially floating and sinking pipeline at authorized pre-determined locations at least 400m outside of the navigation channel. Discharged sediments consisted almost entirely of silt and clays at all three locations.

### Fish sampling

Although not conducted in the James River, underwater sounds emitted by CSDs have been characterized elsewhere [16–18]. Reine, Clarke, and Dickerson [19] describe the multiple sounds associated with CSD operations. Clarke, Dickerson, and Reine [20] measured sounds produced by a large CSD conducting maintenance dredging in Mississippi Sound. Reine and Dickerson measured sounds produced by a medium-sized CSD performing maintenance dredging at the Port of Stockton, California. Based on these characterizations, CSD sounds were categorized as continuous rather than impulsive, with most of the acoustic energy falling in the 70 to 1,000 Hz range. In a 2023 review, Popper and Calfee [21] reported that sturgeon of the genus *Acipenser* could probably detect sounds as low as 50 Hz to slightly above 1,000 Hz, a range that includes most low frequency dredging sounds. The precise distance that juvenile sturgeon would detect sounds is not predictable given available data but can reasonably be assumed to be less than 200 to 300m.

**Fig 2:**
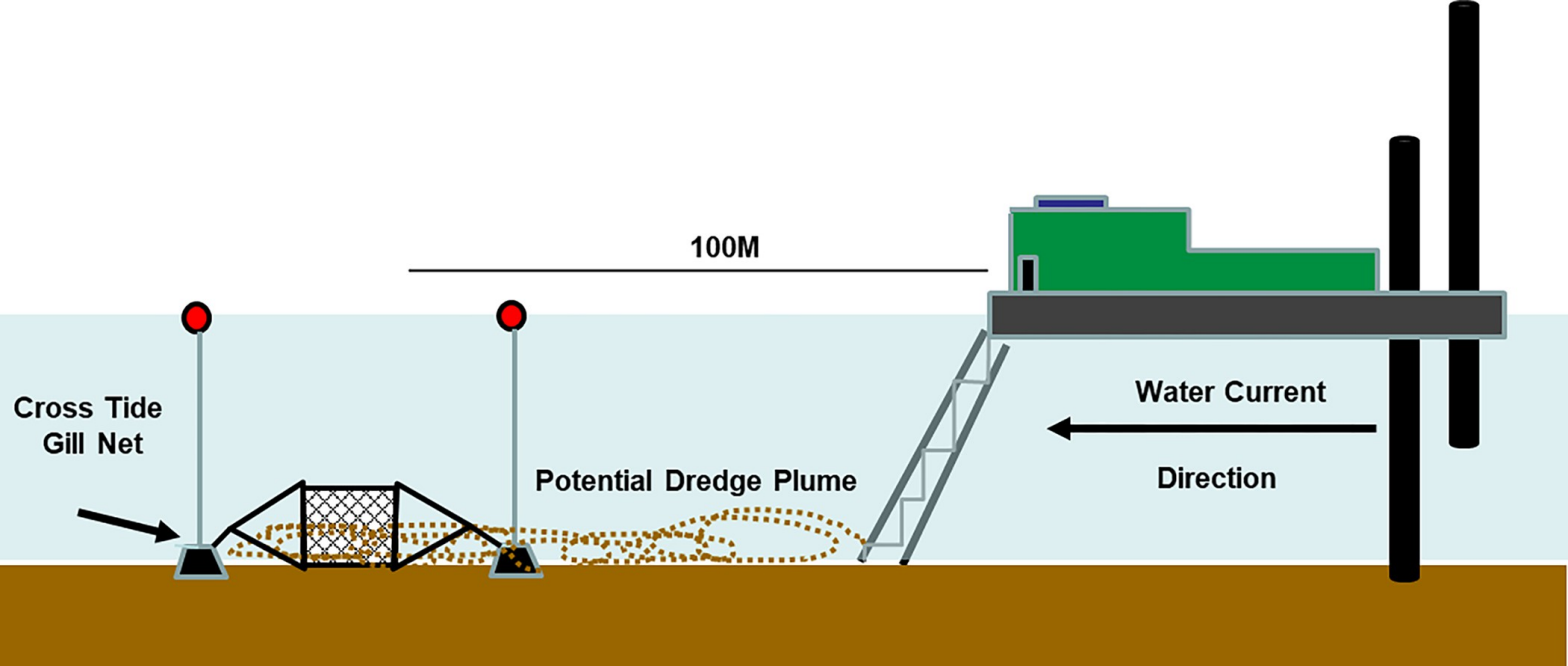
Schematic layout of a gill net deployment (not drawn to scale).

To elucidate the impacts of CSD sounds on juvenile ATS, gill nets were set perpendicular to the channel axis around 100m down current of the dredge barge (Fig 2). The distance was selected because it was close enough that the juveniles should hear the dredge but far enough away if problems occurred during net deployment or retrieval the net/boat would not swing into the CSD. Distance from the dredge was measured with a Leupold RX-800i TBR rangefinder. The position of the cutterhead on the bottom was estimated to be 5m in front of the dredge’s bow with some slight variation due to changes in the ladder angle during normal operation. When set down current of the bow, the gill net was about 95m from the cutterhead, whereas the net was about 145m from the cutterhead when set behind the stern.

Net deployments were conducted during changing tides [22–24] with a maximum net soak time of 1hr. Sets were one hour and typically about 15-30min before to 15-30min after slack current when the water was flowing towards the active dredge. One set at Sandy Point was reduced to 40min due to an approaching ship the crew did not see due to fog. CPUE was ATS caught in the net during a tide change per hour which is equal to the number caught except for one set at Sandy Point. A single gill net consisting of 30m mesh panels of 7.6cm, 10.2cm and 12.7cm stretch mesh was used for the study. All panels were 1.8m tall and attached in a random order for each net set. A linear regression was run on Goose Hill CPUE to see if it changed over time while the dredge was working.

Nets were set in this method to also see if juvenile ATS avoided the turbidity plume created by the cutterhead digging into the substrate along with the sounds generated by the digging process. The plume would have been traveling through the sampling area for the duration of the flood or ebb tidal currents which is at least four hours in the James River, Virginia. All three locations dredged during this study are characterized by relatively naturally high background turbidities and suspended sediment concentrations, which would be slightly elevated by the digging activities [25]. Discharge plumes at in-water placement areas have greater dredge-induced turbidities and suspended sediment concentrations [25], but placement areas are located outside of the channel boundaries in water less than 3m deep.

Upon capture, ATS were immediately placed in a 416L tank that had river water constantly flowing through. Captured ATS were scanned for a passive integrated transponder (PIT) tag. If no PIT tag was detected, a PIT and T-Tag was injected under the dorsal fin. ATS FL was measured in cm and a fin clip was taken from the pelvic fin for genetic analysis. All fish were released at the sampling location after processing which always coincided with water currents moving toward the dredge.

**Fig 3:**
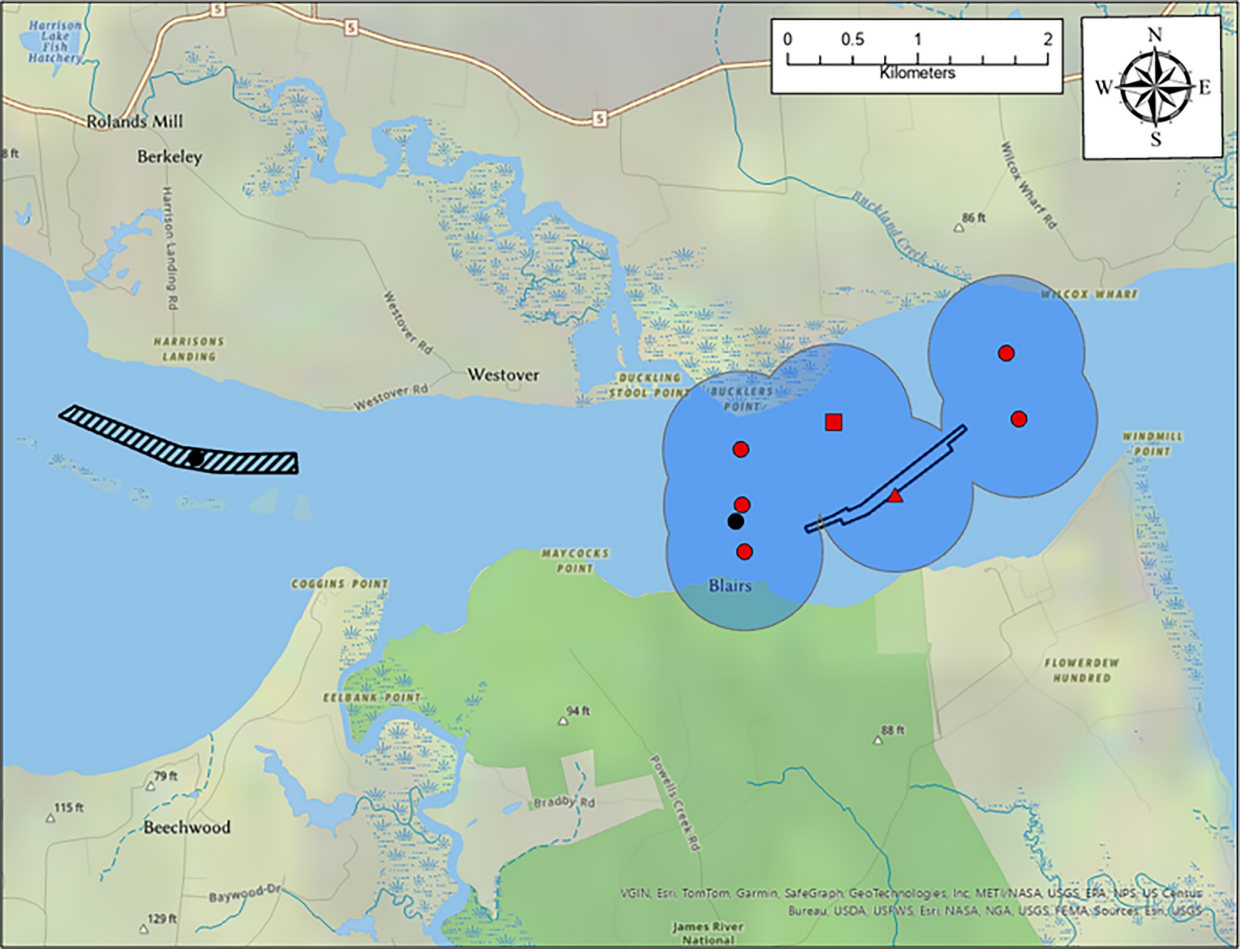
Map showing the dredge prism (black open polygon) and receiver locations at the Windmill Point study area. The black hatched polygon is the reference trawl sampling location. The black points are long-running passive receiver locations while the red points are receiver placed specifically for this study. The red triangle represents the receiver attached to the dredge while the red square is the receiver attached to the sediment discharge pipe. Both the dredge and discharge location shifted during the study with dredge moving throughout the dredge prism and the discharge staying to the north in shallow water. Shaded circles around the red points represent the estimated tag detection range (600m) for the acoustic passive receivers.

Trawl sampling only occurred at Windmill Point because by the time Dancing Point and Goose Hill sampling occurred, ATS had attained a size that rendered currently used trawling methods ineffective. At the Windmill Point study area, a 4.9m wide SRT benthic trawl with 76cm X 38cm doors designed by Innovative Net Systems was towed with the tidal current alongside the dredge and at a reference location about 5km upstream of the dredge. The reference location was selected to ensure that samples were not influenced by any turbidity plume or sound associated with the dredge (Fig 3). The trawl was towed by a single 30m towline connected to a bridle with 8m warp lines to the trawl doors. Tow speeds were 3.0–4.0km/h and started approximately 200m down current and stopped 200m up current after making an arc pattern around the dredge. The trawl tows were within the navigation channel about 30-40m away when passing by the dredge. Two tows were made during each trawl sampling day, one alongside the dredge and one at the reference location. The dredge tow was completed first and the tow time recorded. The tow time started when the 30m towline was fully taut and ended when trawl retrieval began. The same tow procedure and time was then repeated at the upstream reference location and CPUE was the number of ATS captured per tow. Trawl durations were approximately 7–8 minutes. ATS were released where the trawl was retrieved and processed exactly as described above for gill net captures.

### Telemetry

ATS were anesthetized using electronarcosis and had a V7 or V9 Vemco telemetry tag surgically implanted using established protocols [26]. The V7 telemetry tags with a pressure sensor providing depth information had a battery life of 214 days as compared to 912 days for V9 non-pressure tags. The V7 tags had a 130-230sec burst frequency while the V9s tags had a burst frequency or 20-40sec for the first 30 days then changed to 130-230sec for the remainder of the battery life. No more than three ATS were tagged during a single gill net sample. All ATS were released at the sampling location when the water current was moving towards the cutterhead. No ATS captured in trawl samples had telemetry tags implanted.

Telemetered ATS were tracked throughout the tidal portion of the James River using a Vemco passive receiver array (Fig 1). Additional receivers were placed around the Windmill Point study site from November 4^th^ to December 10^th^, 2019 (Fig 3). At Windmill Point, a receiver was attached to the stern of the dredge and another on the discharge pipe about 10m from where sediment was being placed. Range tests estimated that under prevailing weather conditions, the telemetry tags had a detection range of 600m but detections up to 1km did occur. Specific ranges tests were not conducted on the receivers attached to the dredge or discharge pipe, so sounds generated by the dredge operations may have masked signals and thereby decreased the effective detection range. However, measurements of sounds produced by CSDs working in silt/clay sediment indicate that these are relatively quiet such that an assumption can be made that detection range is not severely affected [27,28]. A receiver was attached to the dredge at Dancing Point and Goose Hill but additional receivers were not available at the time to supplement the passive array around the dredge area. The receiver on the dredge at Goose Hill was lost so no detection data from the dredge are available at that location. Detection range tests were not conducted at either Dancing Point or Goose Hill, but the detection range is presumed to be similar to measurements at Windmill Point.

## Results

### Windmill point

**Fig 4:**
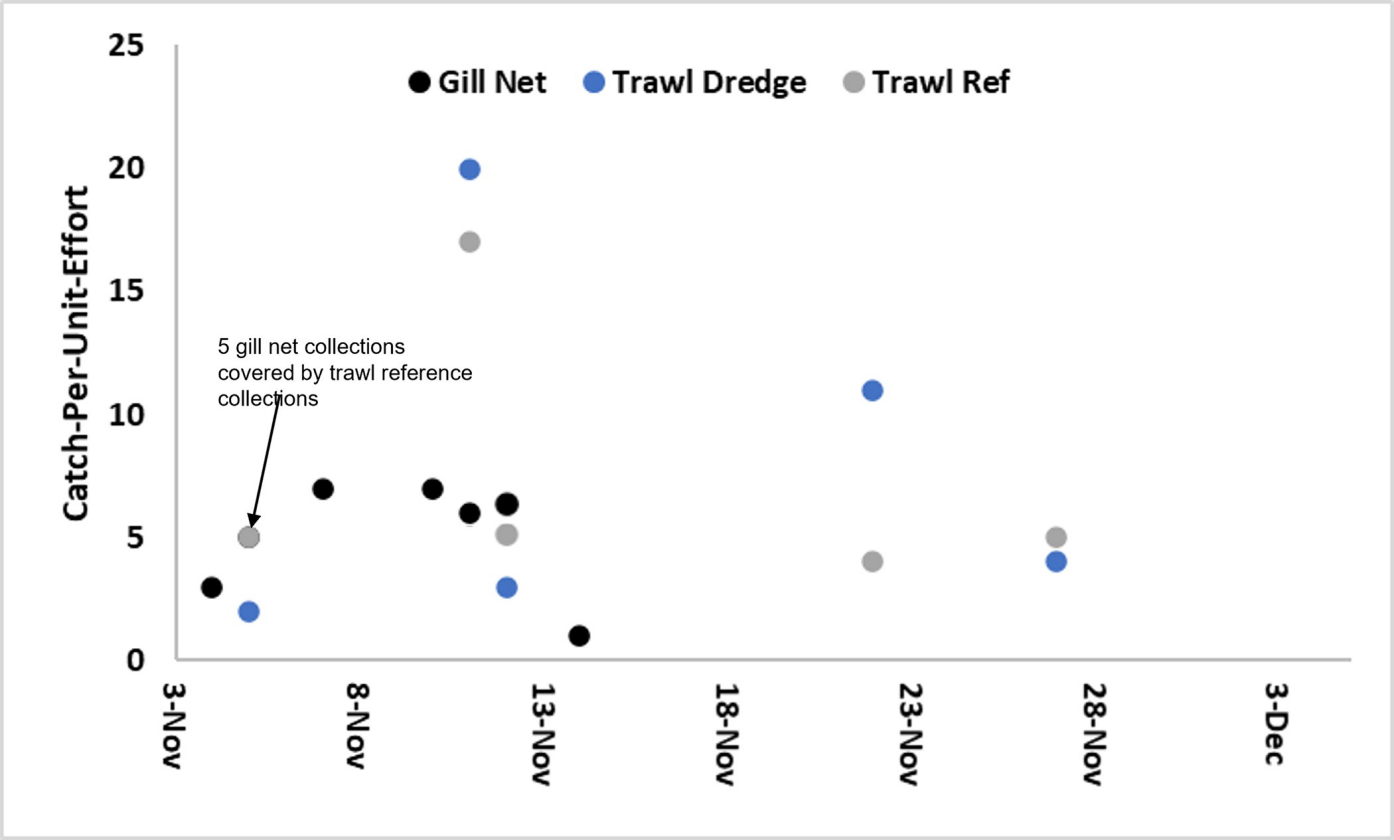
Trawl and gill net catch-per-unit-effort of ATS at the Windmill Point dredge and reference areas. Note that five ATS gill net collections on November 5^th^ coincided with five reference trawl captures from the same day.

Windmill Point dredge operations removed 129,458m^3^ of sediment from November 4^th^ to December 5^th^, 2019. The river width perpendicular to the direction of the navigation channel varied from 1.6–2.2km along the area dredged. The river’s bathymetric cross-section at the dredge area is typical of a confined river channel, with lateral 2-3m deep mudflats sloping downward to the federal channel. Seven gill net sets between November 4^th^ and 14^th^ captured 34 individual ATS ranging from 30-47cm FL. There were no recaptures. Four sets upstream of the dredge captured 21 ATS and downstream sets captured 13 ATS (Fig 4). These ATS are estimated to be 1yr of age.

**Fig 5:**
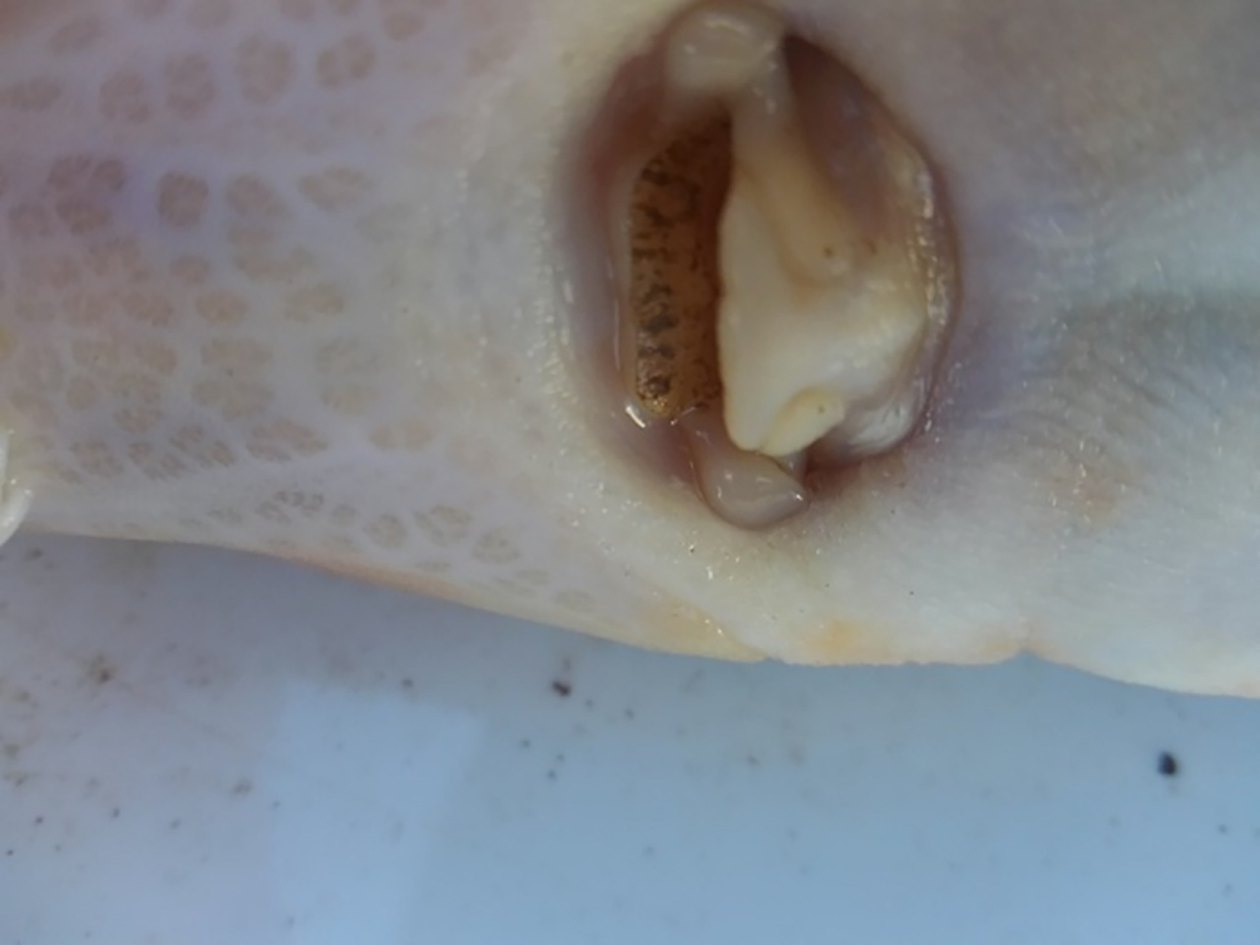
Image of muddy material in the oral cavity, suggesting recent feeding behavior by a juvenile ATS captured during gill net sampling. Photo Credit: VCU Rice Rivers Center.

Age 1-2y juvenile ATS typically feed on benthic invertebrates. To feed upon these benthic invertebrates, ATS swim along the bottom while protruding their mouth onto the benthic substrate. The jaw protrusion creates a suction force that pulls both nourishment and sediment into the mouth [29]. Sediment is often found in the intestines and stomach of ATS [30] and researchers noted sediment in the oral cavity of ATS and shortnose sturgeon *A*. *brevirostrum* that coincided when ingested material was within the stomach during gastric lavage (William “Bill” Post, personal communication, South Carolina Department of Natural Resources). Observations of captured ATS revealed that almost all had sediment material within their oral cavity (Fig 5). Considering the mechanism of how ATS feed, it is reasonable to presume this is a sign of feeding behavior. The research team was not comfortable conducting gastric lavage to verify recent feeding; however, based on the location of the sediment, feeding would be the most rational explanation. From November 5^th^ to the 27^th^, five paired trawl tows captured an additional 71 individual ATS ranging from 31-47cm FL (Fig 4). The dredge trawls captured 39 ATS and the reference trawls captured 32. A two-tailed student t-test indicated no significant difference (p = 0.45) in CPUE between the reference and dredge trawl areas.

**Fig 6:**
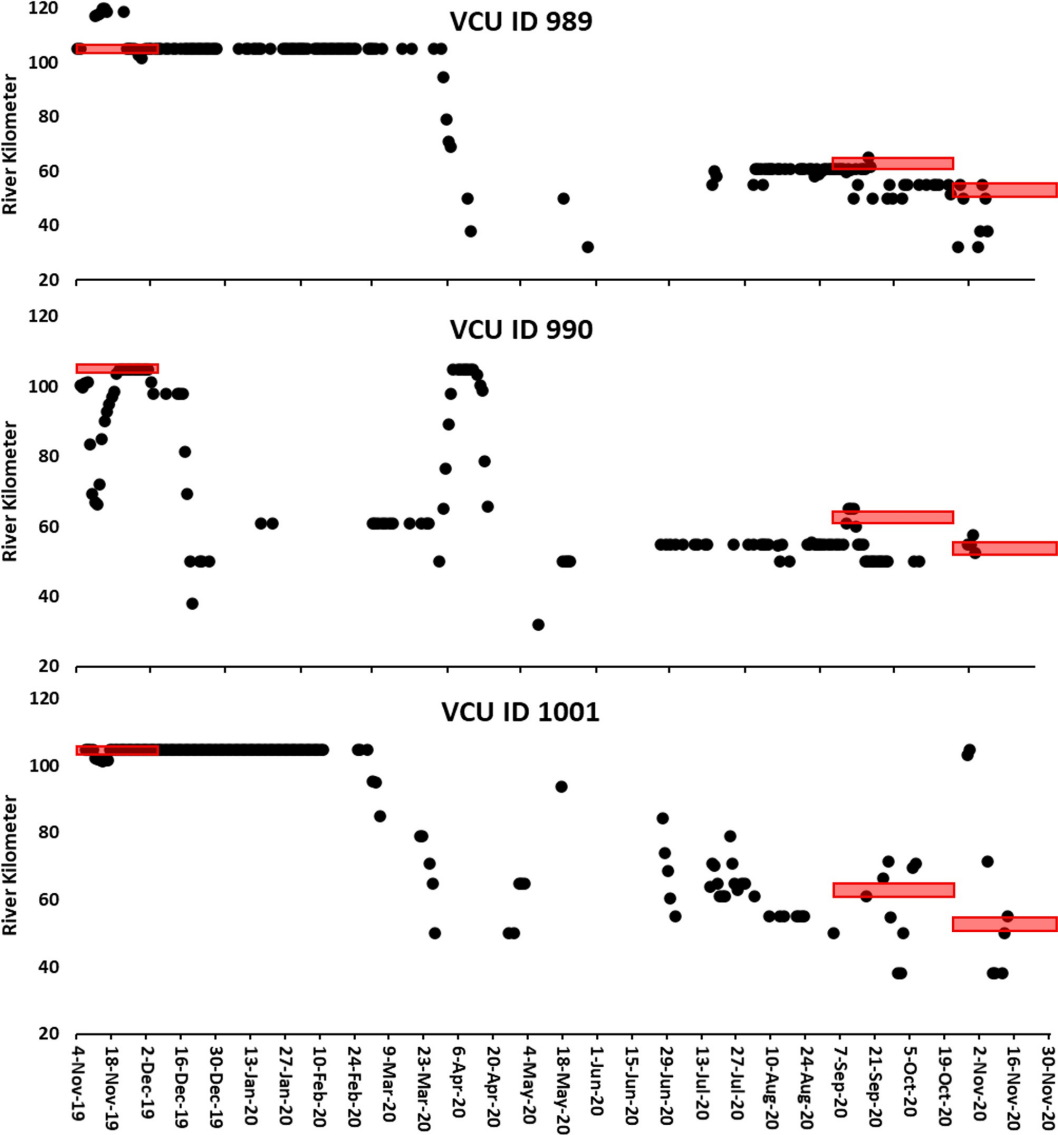
Telemetry examples of a fish moving upstream (VCU 989), downstream (VCU 990) and staying at the tagging location (VCU 1001). The red rectangles symbolize where and when the dredge was working.

**Fig 7:**
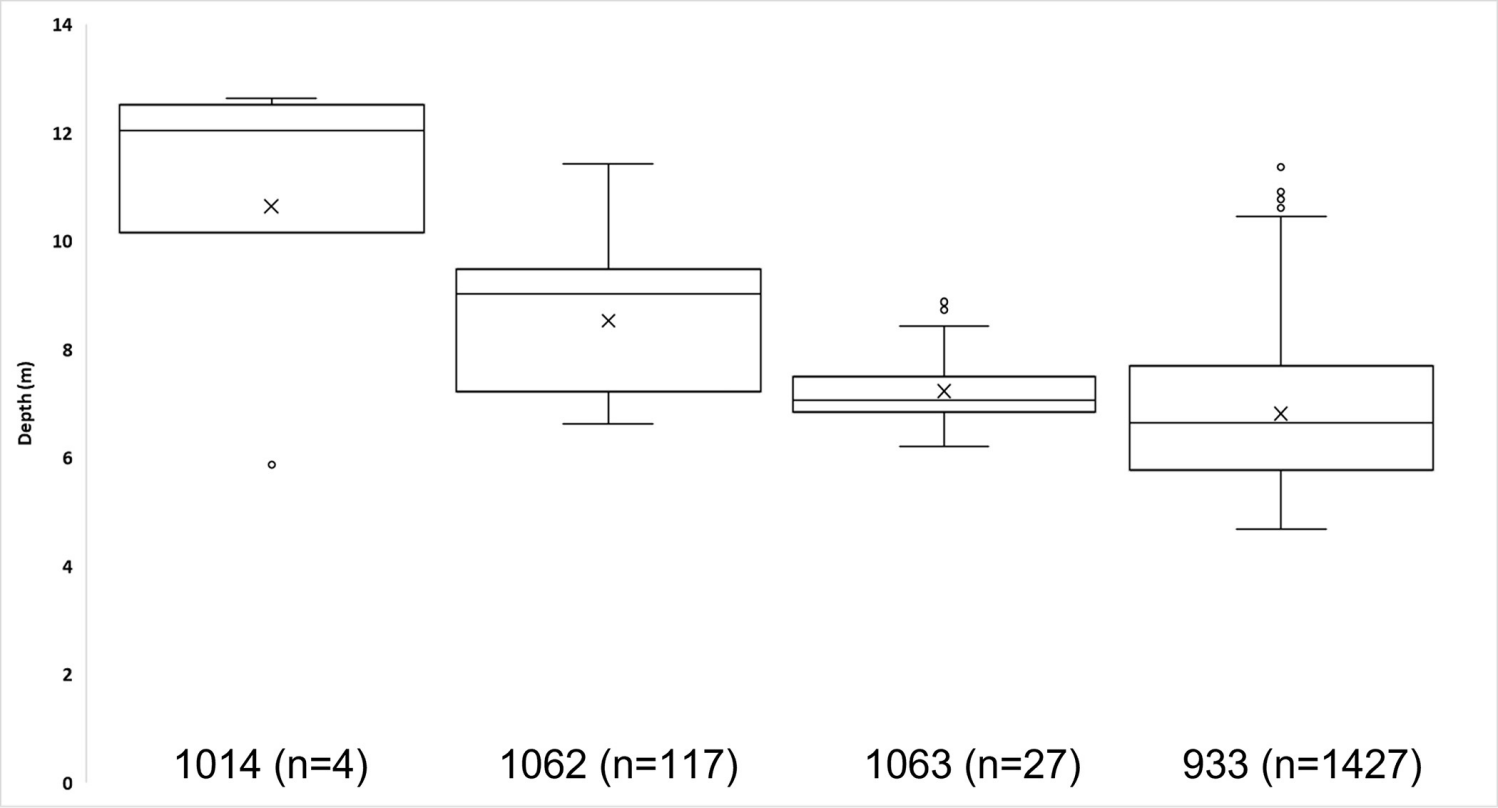
Depth data derived from four tagged juvenile ATS, depicting extent of vertical movements revealed by repeated detections (n = 4, 117, 27 and 1427 respectively).

Eighteen of the juvenile ATS captured in the gill nets were telemetered and released during Windmill Point sampling (Tables 1 and S1). There was disparity in initial movements upon release. Several ATS immediately moved downstream or upstream after being tagged while others stayed within the dredge area (Fig 6). Three additional telemetered ATS tagged over a month prior to the present study moved into the study area during dredge operations. The 21 tagged ATS were detected within the dredge telemetry array from 1–23 separate days totaling 199 days in the dredge array (S1 Table). Tagged ATS passed the dredge a minimum of 52 times without incident with one ATS accounting for 17 passes (Tables 1 and S1). Data from the depth tags suggested that juvenile ATS spent most of the time and at depths that are only available within the navigation channel (Fig 7). The depth tags showed the fish passed the dredge within the channel, meaning fish were likely passing less than 75m of the dredge.

**Table 1:** Summary of telemetry data at three study locations in the James River, Virginia. Detailed data for each location are available in supplementary tables. Tagged juveniles reflect ATS tagged prior to the study and newly tagged individuals. The number of different days detected is the number of calendar days tagged ATS were detected by the receiver attached to the dredge.

LOCATION	TAGGED JUVENILE STURGEON IN ARRAY	RELEASED	NUMBER OF DIFFERENT DAYS DETECTED	DREDGE PASSES
		UPSTREAM–DOWNSTREAM	TOTAL (RANGE)	TOTAL (RANGE)
WINDMILL POINT	21 (18 NEW)	13–6	199 (1–23)	52 (0–17)
DANCING POINT	17 (4 NEW)	9–8	49 (1–16)	31 (0–5)
GOOSE HILL	23 (7 NEW)	16–7	NA	42 (1–6)
				125 TOTAL PASSES

**Fig 8:**
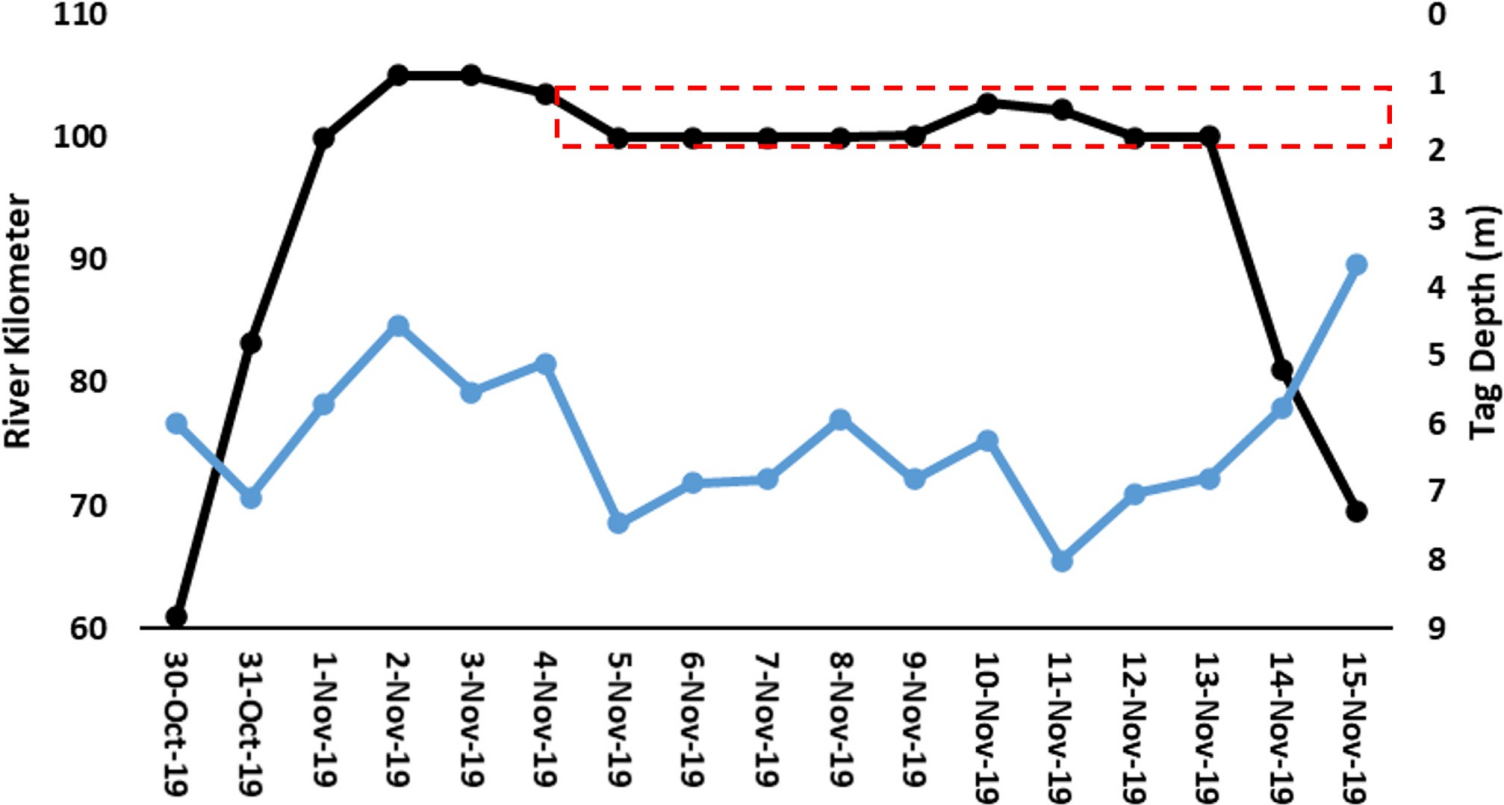
Daily average river kilometer location (black points, left axis) with daily average depth (blue line, right axis) of VCU ATS ID 933. The red dashed rectangle shows the river kilometers where dredging was occurring.

The immediate post-tagging movements are likely influenced by the tagging process. The three telemetered juveniles tagged upstream more than a month prior to this project moved through the study area and were detected by the receiver on the dredge. The best example of presumed normal behavior was ATS 933 which was tagged 36 days prior to the start dredging and was within the dredge array when dredging started on November 4^th^. ATS 933 remained within the dredge array until moving downstream on November 14^th^. During the 10-day period, this juvenile was detected by the receiver attached to the dredge on nine successive days and moved passed the dredge a minimum of three times (Fig 8).

### Dancing point

Dredging at Dancing Point removed 220,219m^3^ of sediment from September 4^th^ to October 22^nd^, 2020. The river width perpendicular to the axis of the navigation channel varied from 4.9–5.7km along the area dredged. The bathymetric cross-sectional profile here consists of a broad river channel with gradual slopes rising to lateral flats with a depth of 2-5m. Gill net sampling started on October 11^th^ and ended on October 21^st^. Five upstream sets and one downstream set captured 33 and 3 ATS, respectively, ranging from 50-64cm FL **(**Table 1). Four telemetry tags were deployed on the upstream side of the dredge (S2 Table). Information gathered from these four tagged ATS was limited as dredging was completed within four days of the ATS being released. As observed at Windmill Point, sediment was visible within the oral cavities of several ATS when removed from the net.

Of the 15 telemetered juvenile ATS still inhabiting the James River, thirteen (87%) were detected at the dredge and/or confirmed moving past the dredge (S2 Table). The previously tagged juveniles had been tagged roughly a year prior, suggesting their movements were no longer influenced by handling during netting and tagging process. The 13 telemetered ATS were confirmed moving past the dredge 28 times; however, due to the distance of the passive receivers upstream and downstream of the dredge, the number of passes was likely greater. The 13 ATS that were at large for about a year were cumulative detected 43 days by the receiver attached to the dredge (Tables 1 and S2). The four ATS tagged at Dancing Point were detected six days by the dredge receiver and moved past the dredge at least three times (Tables 1 and S2).

There were six instances when ATS passed the dredge but were not detected by the receiver attached to the dredge. Non-detection by the dredge receiver may reflect passage at a distance beyond receiver detection in this wide river section or simply masking by dredge sounds. Only one ATS (VCU ATS ID 933) with a depth sensor tag was detected at Dancing Point. Depths for this ATS ranged from 1.0–6.9m deep, suggesting that the fish could have remained outside of the navigation channel even when detected by the receiver on the dredge.

### Goose Hill

**Fig 9:**
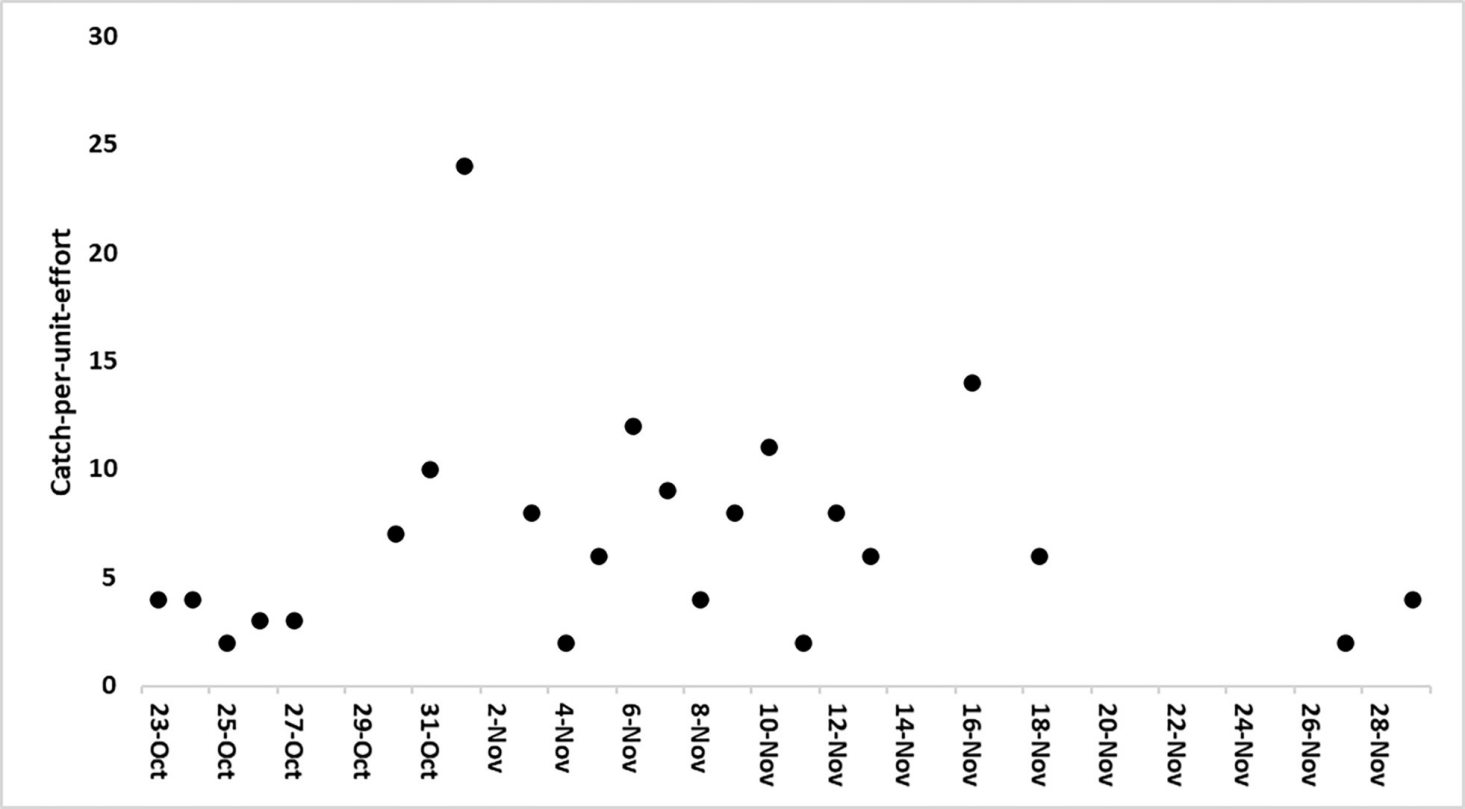
Catch-per-unit-effort at Goose Hill during the dredge period. Linear equation (Y = 0.0018x-70.58, r^2^ = 0.00001, df = 22, p = 0.99).

Dredging and gill netting occurred at Goose Hill from October 23^rd^ to November 29^th^. Dredging removed 164,890m^3^ of sediment from the channel. The river width perpendicular to the axis of the navigation channel varied from 4.3–5.1km along the area dredged. The river cross-section bathymetry resembles that of Dancing Point. Eight sets downstream of the dredge captured 48 ATS while 15 upstream sets captured 111 ATS ranging from 45-71cm FL (Fig 9). There were two instances when an ATS was captured twice during the Goose Hill sampling. Both were first captured on November 10^th^ and recaptured on the same side of the dredge the following day. A linear regression found no notable CPUE trends over the time of the sampling period (Fig 9). As observed at Windmill Point and Dancing Point, sediment was noted in the oral cavities as ATS were removed from the net.

Seven ATS ranging from 51-57cm FL (Tables 1 and S3) were tagged at Goose Hill. Due to the receiver on the dredge being lost and the closest passive receivers were located several km upstream and downstream from the dredge, close proximity tracking to the dredge was not available. However, based on detections at the distant receivers, the seven ATS tagged at Goose Hill moved past the dredge a minimum of 13 times. Similar to Dancing Point, there were 13 ATS tagged roughly a year earlier detected in the passive array around Goose Hill. These juveniles were confirmed to have passed the active dredge a minimum of 24 times (Tables 1 and S3). Additionally, three of the four ATS telemetered at Dancing Point passed the active dredge a minimum of five times (Tables 1 and S3). Due to sparse receiver coverage at the area, the number of times ATS moved past the active dredge was probably greater.

## Discussion

Concerns persist that navigation dredging operations in rivers may impact migratory fishes, among them ATS. Tidal portions of rivers along the Atlantic coast are most frequently dredged by CSD. This mode of dredging entails embedding a rotating cutter in the sediment bed, an action that forms a sediment/water slurry which is hydraulically pumped through a pipeline to a designated discharge site, either upland or in-water. Descriptions of the cutterhead actions are given by Hayes et al. [31] and Henriksen et al. [32]. In the James River, Virginia, required periodic maintenance dredging is typically performed typically in this manner, with discharge back into the river outside of the navigation channel boundaries. With respect to ATS, categories of concern linked to this particular dredging process include habitat alteration, disturbance of benthic resources that represent forage items, hydraulic entrainment, and impediment or blockage of movements. Concerns related to impediment or blockage of movements stem from hypothetical responses to turbidity/suspended sediment plumes created by the dredge or to sounds emitted by the dredge. The focus of the present study is on the latter topics; gathering data of mortality of juvenile ATS due to entrainment into the cutterhead’s intake vortices, evidence that juvenile ATS avoided an active dredge site and evidence if juveniles would move past active dredge operations.

### Hydraulic entrainment

Concerns that CSDs could entrain and thereby injure or kill diverse aquatic organisms have proven to be difficult to confirm or refute [8]. Attention has largely focused on sea turtle and sturgeon entrainment by TSHD, which are frequently employed to maintain harbor entrance channels. Long-term systematic monitoring of take by TSHDs in the United States has documented that sturgeon can be entrained. CSD projects are not systematically monitored for take and CSD and TSHD processes are very different. CSDs are relatively stationary whereas TSHDs are mobile. A great deal of effort has been invested in systematic monitoring of sea turtle takes by TSHDs and research into dredging project management practices to reduce takes [33]. However, due to difficult logistical challenges in detecting takes by CSDs, investigators have largely relied on assessments of risk of entrainment based on knowledge of the dredging process and capabilities of the target organisms to behaviorally avoid or physically escape entrainment.

Several studies have investigated the swimming capabilities of sturgeon [34], including members of the genus *Acipenser* [35–38]. Most of these studies measured both endurance and critical burst swimming speeds in flow fields of various velocities, frequently with the objective of informing the design of fish passage structures. Other studies have specifically addressed sturgeon swimming capabilities with respect to risk of entrainment by dredges [39–41]. Hoover et al. [39] presented a conceptual model for determining risk of entrainment considering species specific factors including rheotaxis, sustained swimming capability, and burst swimming speed, which they treated as an escape speed. By comparing estimated CSD intake velocities of 50cm/s at 1.5m from the cutterhead to a measured escape speed in tandem with measures of swimming performance and behavior, they calculated susceptibility to entrainment. Juvenile Pallid Sturgeon (*Scaphirhynchus albus*) escape speed was estimated to be 51-70cm/s, which when considered with other behavioral traits led to a conclusion that they would be slightly susceptible to entrainment if they came within the influence of the intake flow field. Boysen and Hoover [40] used swimming performance to estimate risk of entrainment for trained and untrained juvenile White Sturgeon. Untrained White Sturgeon juveniles (80-82mm) had minimum escape speeds of 40cm/s, although they noted that escape speeds varied widely among individuals. Juvenile White Sturgeon “trained” by exposure to continual water flows of 10-12cm/s for extended periods had substantially higher escape speeds. Trained fish were considered to be representative of wild rather than hatchery-reared juveniles. Hoover et al. [39] used a similar approach to evaluate susceptibility to entrainment of juvenile Lake Sturgeon and Pallid Sturgeon from different populations. They concluded that the risk of entrainment for all tested cohorts was minimal unless they entered a 1.25m radius of a dredge intake flow field. Results of the above studies are consistent with results of the present study in that tracking data indicated that telemetry tagged 1-2yr juvenile ATS moved past an active cutterhead at least 125 times without impingement/entrainment.

### Turbidity or suspended sediment plumes

One caveat identified by the authors of the entrainment studies described above was that in the field, additional factors could affect the outcome of dredge encounters that were not addressed in their investigations. For example, in the field, sturgeon might behave differently in the presence of suspended sediments or certain dredging-induced sounds. Wilkens et al. [37] investigated survival and swimming performance of juvenile ATS (10-17cm FL) exposed to suspended sediment concentrations of 100, 250, and 500mg/l for three days prior to swimming tests. Of 90 juveniles tested 86 (96%) survived. One fish was lost at 250mg/l and 3 at 500mg/l. Critical swimming speeds were evaluated to be moderate at 21 to 31cm/s. They concluded that the lack of substantial immediate impacts on mortality or swimming performance in the laboratory suggested that free swimming fish in the field would experience minimal ill effects from dredging operations. In regards to this field study, the juvenile ATS were not confined to but inhabited an area around 100m down current of dredge operations, suggesting dredge operations did not generate too stressful of an environment.

### Underwater sounds

Potential impacts of anthropogenic sound, including sounds produced by dredges, on aquatic organisms have received heightened attention in recent years. Although largely focused on the effects of intense impulse sounds, such as those associated with pile driving or underwater explosions, more subtle effects of chronic exposure to lower intensity sound on behavior and physiology have also been identified. Relatively few characterizations of sounds produced by various modes of navigation dredging have been published. Coupled with a general lack of knowledge pertaining to the hearing capabilities of many aquatic organisms and their threshold responses to sound exposures, informed management decisions regarding dredge operations in the presence of potentially susceptible organisms are handicapped. Sturgeon represent a prime example of the need for a better understanding of their hearing capabilities [18].

## Conclusions

During the present study, 268 age 1-2yr old ATS were captured in close proximity to an active CSD. Receiver detections showed that 31 acoustically tagged ATS passed dredge operations a minimum of 125 times. Based on detections of all tagged juveniles following the cessation of dredging, this cohort of individuals showed 100% survival. ATS showed no indication of an avoidance response even when within 100m of the cutterhead where they would be exposed to dredging-induced suspended sediments and sounds. Sediment in the oral cavities of many netted ATS suggests that ATS were feeding when caught. Juvenile ATS were still being caught towards the end of all three dredge operations even when dredging had been ongoing for over a month. If sound or plumes from the dredging created stressful conditions within 100m of the dredge, it is unlikely that highly mobile juveniles would be caught so close to the dredge.

This study could have been improved with better receiver coverage/VPS, setting a coinciding gill net up current during down current sets, use shorter nets so sampling could occur closer to the CSD while maintaining safe practices, or doing a Before-After-Control-Impact study around the dredge and in-water placement areas. Increased numbers of telemetered juvenile ATS would have improved the results; however, the sample size for this study is comparable [22] or more than double [24,42] the tag numbers used to infer population behavior within other river systems. These improvements could better elucidate on whether the dredge may act as a deterrent or attractant, does the dredge play a factor in juvenile movements, or if juveniles are entrained by the CSD.

To our knowledge, this is the first study to provide these types of data which decision makers have only been able to speculate. While the study could have been improved, current data show that juvenile ATS survived when swimming past dredge, the dredge did not act as a barrier operations and juveniles did not avoid the immediate dredge area even when free to do so. Although it cannot be said that CSD dredging as conducted in the James River or other rivers poses zero risk of entrainment or impeded movement, the data gathered from this study found no evidence of mortality or blocking movements. However, in tandem the two studies [10] provide strong evidence that the presence of an active CSD and associated sound did not affect survival or impede movements of either juvenile or adult ATS in the James River.

## Supporting information

S1 Table Information for tags deployed and detected at the Windmill Point dredge location. (TIF)

S2 Table Information for tags deployed and detected at the Dancing Point dredge location. (TIF)

S3 Table Information for tags deployed and detected at the Goose Hill dredge location. (TIF)
