# Supplementary figures and images for: Juvenile Atlantic sturgeon survival and movement in proximity to an active cutterhead suction dredge
Source: PLoS One. 2024 Nov 27;19(11):e0300489. doi: 10.1371/journal.pone.0300489 (PMC11602117; doi:10.1371/journal.pone.0300489)

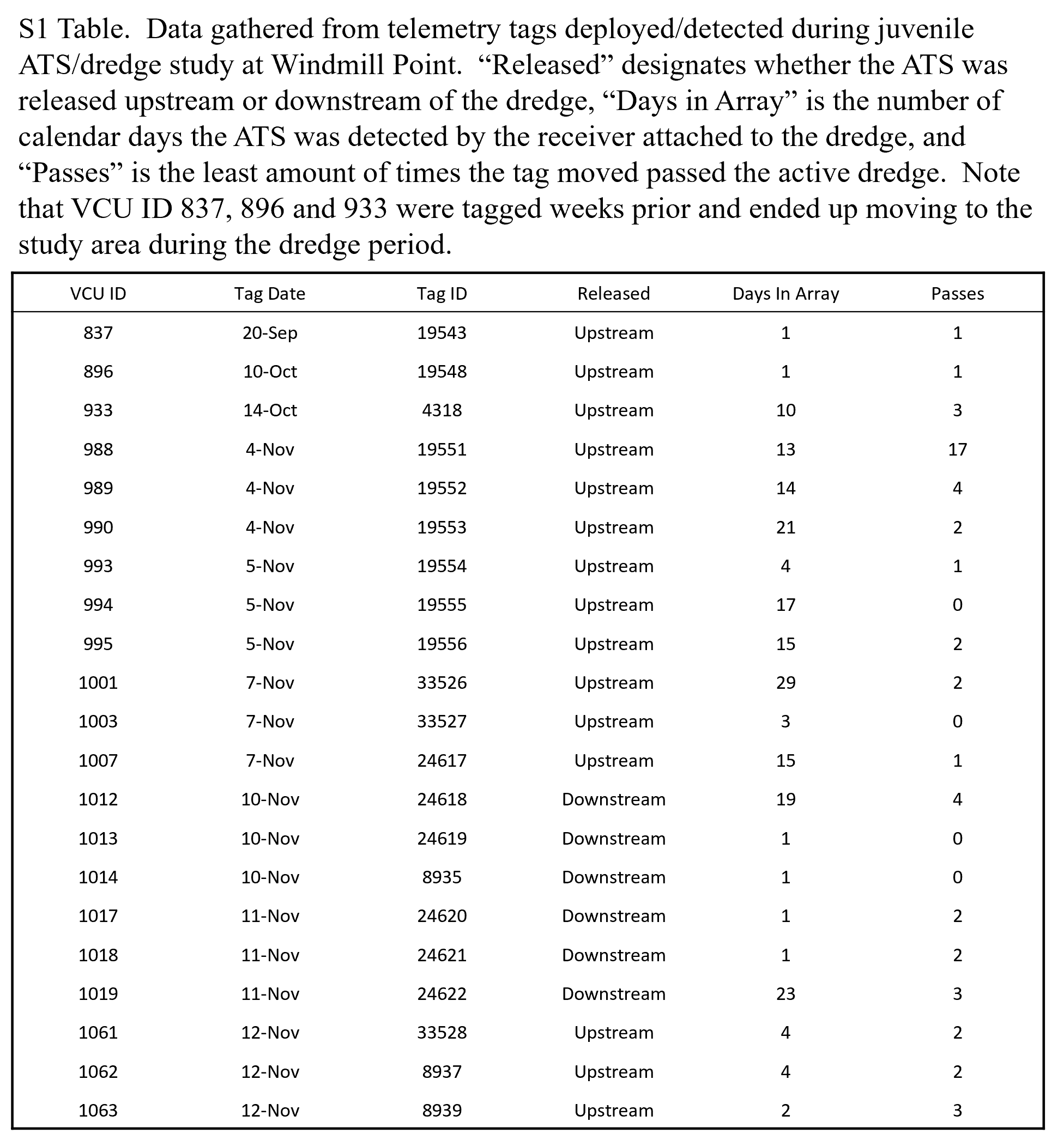

Supplement: S1 Table — (TIF) [file pone.0300489.s001.tif]

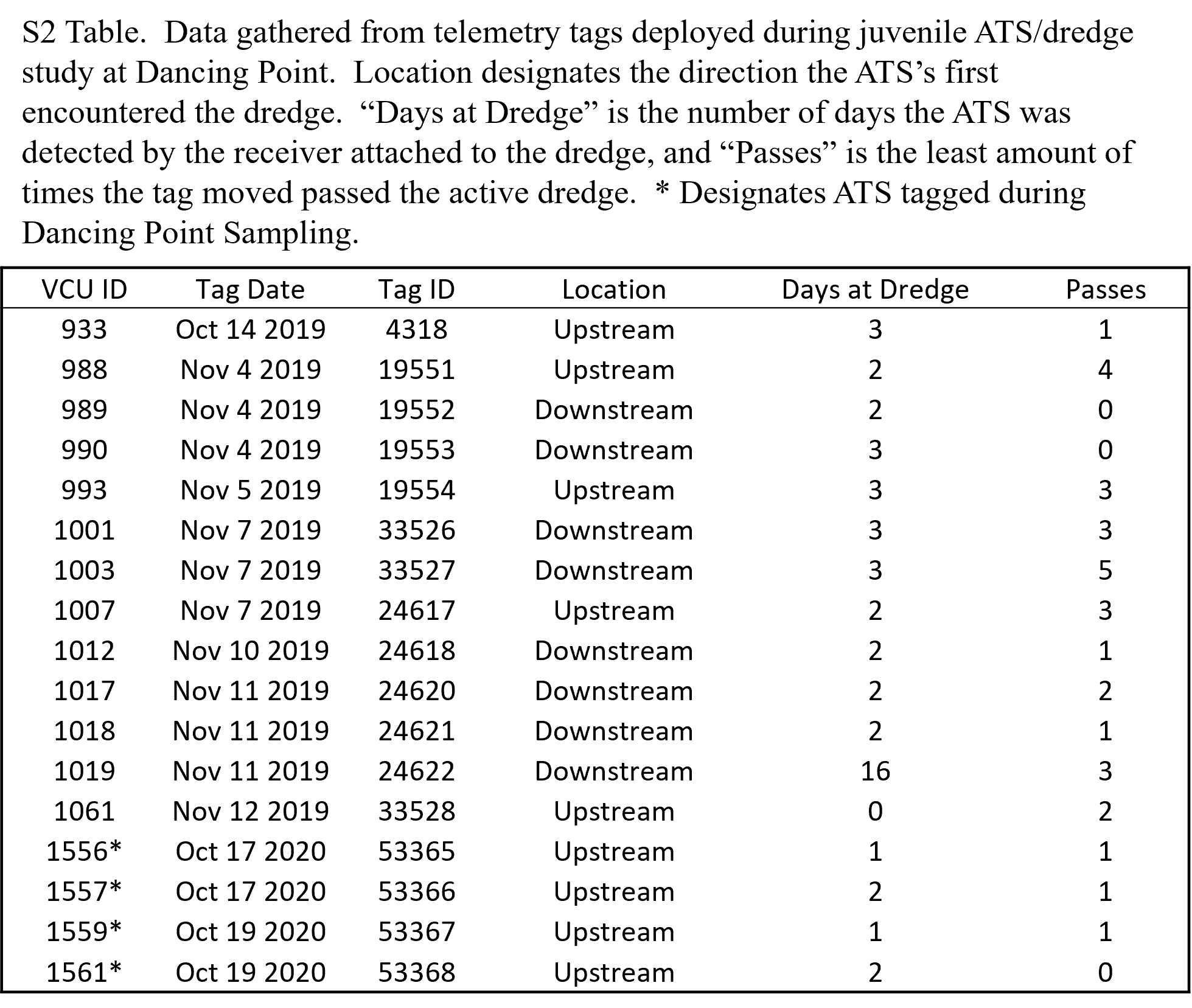

Supplement: S2 Table — (TIF) [file pone.0300489.s002.tif]

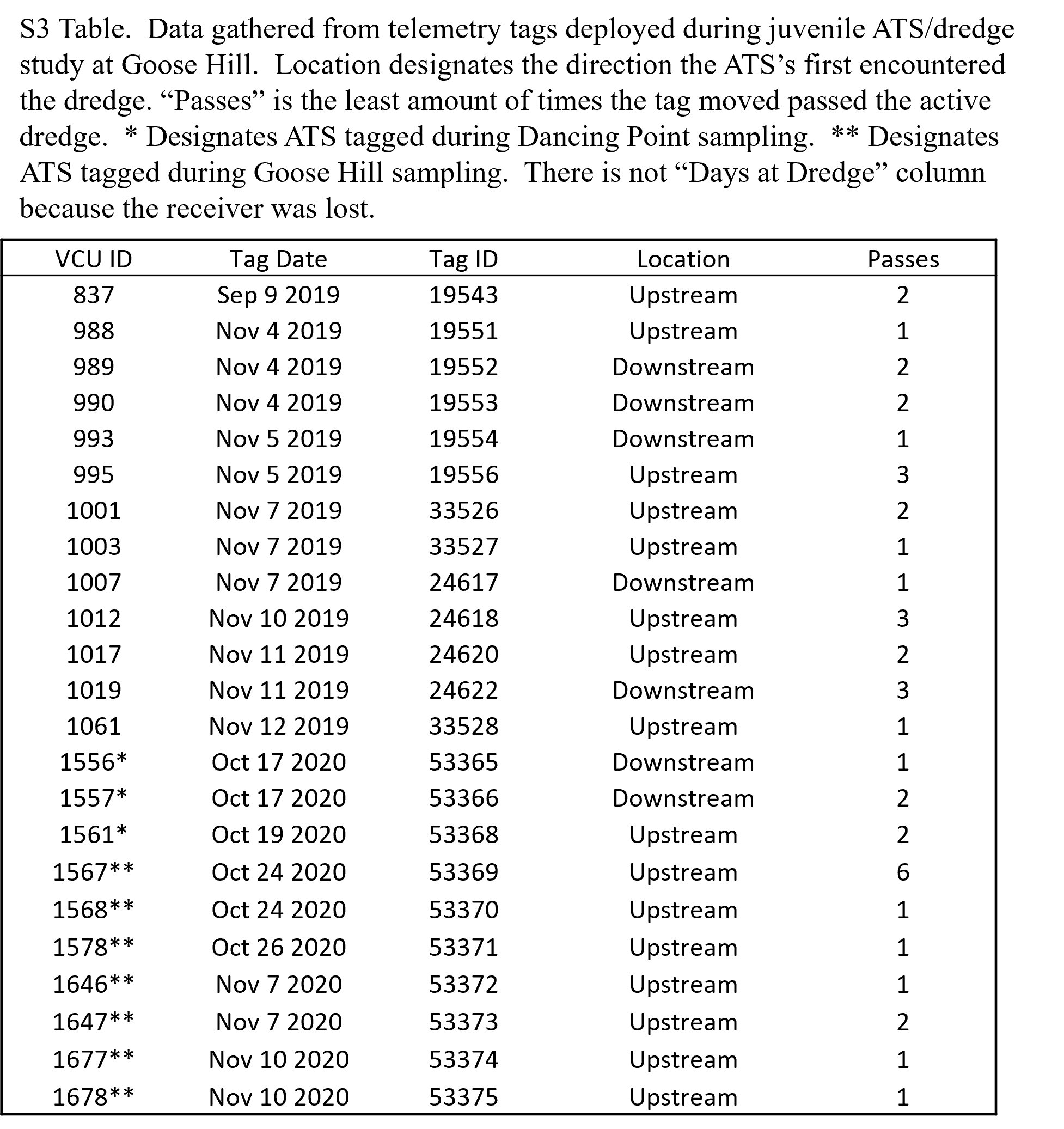

Supplement: S3 Table — (TIF) [file pone.0300489.s003.tif]
